# Influence of the embedded participant on learners’ performance during high-fidelity simulation sessions in healthcare

**DOI:** 10.1186/s12909-023-04724-0

**Published:** 2023-10-11

**Authors:** Ayça Koca, Sophie Schlatter, Quentin Delas, Lucas Denoyel, Jean-Jacques Lehot, Marc Lilot, Thomas Rimmelé

**Affiliations:** 1 Department of Emergency Medicine, Ankara University School of Medicine, Ankara, Turkey; 2 Claude Bernard Lyon 1 University, Hospices Civils de Lyon, Centre Lyonnais d’Enseignement par la Simulation en Santé (CLESS), SimuLyon, Lyon, France; 3 Research on Healthcare Performance (RESHAPE), INSERM U1290, Lyon, France; 4 Department of Anaesthesiology and Critical Care Medicine, Hospices Civils de Lyon, Edouard Herriot Hospital, Lyon, France; 5 Department of Anaesthesiology and Critical Care Medicine, Louis Pradel Hospital, Hospices Civils de Lyon, Groupement Hospitalier Est, Lyon, France; 6 Pathophysiology of Injury-Induced Immunosuppression (Pi3, Claude Bernard Lyon 1 University-Biomerieux-Hospices Civils de Lyon, Lyon, 7426 EA France

**Keywords:** Embedded participant, Full-scale simulation, High-fidelity simulation, Medical education, Non-technical performance, Technical performance, Survey

## Abstract

**Background:**

The embedded participant (EP) plays a key role during a full scale/high-fidelity simulation (HFS) session. He/she is expected to guide the learner towards the achievement of the educational objectives of the scenario. However, his/her influence on learners’ performance stands undetermined and this effect remains briefly addressed in the literature. This study primarily aims to assess whether the EP could have an influence on the performance of the learner during a HFS scenario. The secondary aim was to establish an inventory of the EP practices in France.

**Methods:**

This retrospective study was conducted in Lyon Claude Bernard University Health Simulation Centre (France). Anaesthesia and critical care residents in postgraduate years 1 to 5 who were scheduled for their HFS sessions during the 2016 to 2021 academic years were included. Two investigators independently evaluated the resident performance regarding both technical and non-technical skills from video recordings. In addition, a nationwide survey was sent out by email through the networks of the Francophone Healthcare Simulation Society (SoFraSimS, Société Francophone de Simulation en Santé) to collect information on EP practices in French-speaking Simulation centres.

**Results:**

From a total of 344 HFS videos analysed, a cohort of 19 experienced EPs was identified. The EPs had an influence on the technical and non-technical performances of the learners. The 147 responses to the survey showed that predefined rules of EP good practice existed in only 36% of the simulation centres and 65% of respondents believed that specific EP training would be justified.

**Conclusion:**

The EP can exert an influence on the performance of the learners during HFS. For acting as an EP, a great variability of practices and a lack of training are reported by professionals working in simulation centres. These results suggest that more attention must be paid to EP training and attitudes during simulation, especially if summative simulations are extensively developed.

**Supplementary Information:**

The online version contains supplementary material available at 10.1186/s12909-023-04724-0.

## Introduction

Simulation in healthcare has emerged as a strong and effective teaching tool that allows medical students and healthcare professionals to practice, repeat and manage various clinical situations and environments without causing harm to real patients [1]. Realistic scenarios, which mimic clinical cases that challenge learners, improve safety, quality, and education in healthcare [2]. Combined with advances in technology, simulation-based training using high-fidelity manikins has become a core component of medical education [3]. Importantly, a successful simulation session requires to be well planned with a well-executed scenario, in order to perform an optimized debriefing centred on pedagogical objectives for each scenario. Therefore, embedded participants (EP) are involved to portray roles during the scenario [4]. The Healthcare Simulation Dictionary defines the EP as an individual who is trained or scripted to play a role in a simulation encounter to guide the scenario [5, 6]. Various terms such as “comparse”, “actor” or “confederate” are also used to define the person in charge of the course of the scenario. This person indeed helps to bring out a specific learning objective by providing assistance or guidance that is meant to be indirect or unobtrusive [7]. The EP of a full-scale simulation, also called high-fidelity simulation (HFS), is given responsibility and supervision for the course of the simulation-based experience [4, 8]. His/her role is to help lead the scenario to the correct and wanted direction that is determined in advance according to the pedagogical objectives set by the main instructor of the scenario. He/she promotes the implementation of a realistic environment and ensure learners’ full immersion in the simulated context [9]. EPs are briefed and prepared to avoid unwanted, dangerous events or learners’ frustration during HFS. The EP, who is most of the time in contact with the main instructor *via* headset, is expected to manage learners and issues that may arise during the simulation session such as equipment failure. He/she supervises evolving needs of the participant (e.g., intravenous access, medication, laboratory results) or unexpected behaviours by adapting to the actions and to the level of the learners. He/she manages physical and psychological risks related to simulation to maintain a safe environment [4, 10, 11].

Thus, the EP is one of the most powerful “tools” available to simulation instructors. Intervening at the heart of the action, all of these key roles suggest that the EP may influence the performance of the learners. Surprisingly, despite the extensive orientation of simulation as a summative or normative performance evaluation tool, there is a paucity of data regarding the expertise, contribution, or influence of the EP on the simulation session and its impact on the immediate performance of learners. This study first aims to assess the role of the EP on the technical and non-technical performance of learners during simulated situations. This research also secondarily sought to establish an inventory of the practices of EPs in France through a national survey.

## Methods

### Study design and ethics considerations

This retrospective study was conducted in an academic Health Simulation Centre between December 1, 2020, and June 30, 2021. The study was approved by the Ethics Committee of the French Society of Anaesthesia, Critical Care and Perioperative Medicine (SFAR) (IRB: 00010254-2021-109). The study protocol was registered on March 2021 on clinical trial.gov (Protocol ID: NCT04898660).

### Population and setting

Anaesthesia and critical care residents in postgraduate years 1 to 5 at Lyon Claude Bernard University Hospitals who were scheduled for their HFS sessions at the health simulation centre during the 2016 to 2021 academic years were included. These sessions were part of the compulsory core curriculum of their residency course. For the purpose of research studies, the sessions were recorded, and consent for video recording was obtained from all participants. Various scenarios were simulated, all concerning critical situations involving neonates, children, pregnant women or adult patients. A standard simulation session at the simulation centre included a welcome introduction, a presentation of the simulation room and all of the available equipment and manikins (general briefing). Then, several simulation scenarios were consecutively run, each one of them starting with a specific briefing of the scenario, followed by the scenario by itself and a debriefing. These half-day sessions included four different scenarios on various topics of critical care and medicine and were scheduled on a four-hour period (approximately one hour per scenario), allowing each student to be the active participant of one scenario and an observer of the three others.

### The embedded participants

Each EP was identified from video recordings and was presented with a code composed of a letter and a number; the number represented the number of scenarios facilitated. For example, an EP coded as “A7” indicated that EP A was involved in 7 simulation scenarios. The EPs were instructors of our institution’s Health Simulation Centre, and their role was to portray the assisting nurse. During the scenarios, the EPs knew the scenario and its objectives. While no specific instruction regarding their behaviours was given, the rules of good practice, such as being benevolent and caring, were remembered. They were always equipped with a headset allowing private communication with the lead instructor.

### Performance evaluation

Two investigators independently evaluated the residents’ performance from the video recordings regarding both technical and non-technical skills. Technical skills for each scenario were assessed based on the related international recommendations and guidelines. Predetermined grids were developed by the instructors who created the scenarios. They were helped by local and national experts who shared their views to reach a consensus, taking into account both existing international and national guidelines and recommendations and the specific characteristics of the scenario. As an example, the grid for the epiglottitis scenario is provided as an additional file (Additional file 1). Each item was associated with a number of points so that the total was 100. When the expected action was performed without any suggestion from the EP, all points were awarded for the item. When an action considered essential to the scenario was not performed, the lead instructor requested the EP to suggest it (implicitly or explicitly) through the headset. If the EP implicitly suggested the action to the residents, half of the points were given. No points were attributed if the action was performed following a clear explicit suggestion from the EP or in case the action was not done. The behaviour of the EP was not evaluated along the current study. Non-technical skills were assessed using the Ottawa Crisis Resource Management global rating scale [12]. Ratings of the six Ottawa criteria (global performance, leadership, problem solving, situational awareness, resource utilization and communication skills) were summed, and results ranging from 6 to 42 points were subsequently converted to out of 100. The mean of the scores given by the two investigators was obtained for each participant.

### The survey

To collect the EPs’ demographic characteristics and information regarding simulation experience, a survey was sent out. The survey design followed current guidelines for self-administered clinician surveys [13]. All survey items were reviewed by an expert team of eight professionals including educational experts, EPs, medical doctors, and researchers in simulation for healthcare. For consistency, operational definitions for the terms HFS and EP were provided in the survey introduction. An item survey instrument was designed and grouped into three themes: demographic characteristics, current experience in HFS and characteristics of the simulation centre, barriers and incentives in facilitating a simulation scenario. A 6-point rating scale was used to identify responses, an even number of items in the response scale was preferred to lead respondents to take side in one direction and optimize answers. The final survey was web-based, informed consent for participation was implied with completion of the online questionnaire, after reading the survey introduction (Additional file 2). In addition to the EPs involved in this study, this survey was also addressed to simulation centres in France *via* the support of the French Health Simulation Society (SoFraSimS, Société Francophone de Simulation en Santé) to establish an inventory of the practices of EPs in France. The survey was sent out to 600 simulation instructors. Only questionnaires that were fully completed were included in the statistical analysis (response rate 24.7%).

### Data analysis

To evaluate a representative sample of scenario and habits, the scenarios had to be performed more than five times and with at least three different EPs to be included for further evaluation. Interrater reliability was assessed with the absolute interclass correlation coefficients of performance measures on a random sample of 24 scenarios (package ltm, cronbach.alpha function).

First, the overall influence of the EP on technical and non-technical performances was assessed using Kruskal-Wallis tests. When an overall effect was detected, *post hoc* comparisons were performed to see if the influence of each EP was different from the mean influence of all other EPs using the Wilcoxon-test. Then, a rigid correction for multitesting was applied to the P value using the Holm correction. A p value of 0.05 was considered significant.

Second, characteristics of the EP that may have influenced learners’ performance were determined with linear mixed models. A random effect was applied to EP identity. Fixed effects included gender (Men, Women), training in simulation (yes, no), expertise in simulation (expert, novice), teaching status (yes, no), profession (non-physician, physician) and longer expertise in profession (integral variable). As simulation sessions occurred in different contexts, all models were adjusted for the timing-specific period. The model was applied separately for technical and non-technical performance. The absence of predictor multicollinearity and the residual models were checked. Statistical analysis was performed with R studio (V 4.1.2, R foundation, Vienna, Austria).

Third, the survey demographics were analysed using univariate summaries (means (± SD : standard deviations), medians [25th -75th] and ranges) for continuous variables, and frequencies and percentages were used to summarize categorical variables.

## Results

A total of 344 simulation scenario videos were included in the analysis. The interrater reliability for technical and non-technical performances was excellent (technical: 0.99, 95% CI: 0.97 to 0.99; non-technical 0.94, 95% CI: 0.85 to 0.98). Nineteen EPs were involved, and their number of participations in HFS sessions as an EP varied from 7 to 61 (mean 18 ± 14). Of the 19 EPs, 15 were male and 14 were physicians (74%). A total of 16 underwent a formation in simulation, of which 13 had a university diploma to certify this. Eleven of them were considered “experts” regarding their experience in simulation with an experience of five years or more.

### Influence of EP on learners’ performances

**Fig. 1:**
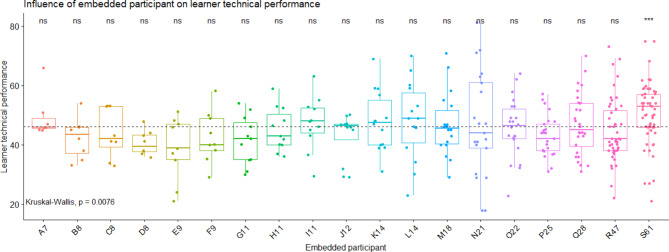
**Learner’s technical performance according to the embedded participant identity**. Each EP is presented with a code composed by a letter and a number, the number representing the number of participations in scenarios as embedded participant. Each point corresponds to one learner’s performance score. The grey dashed line corresponds to the overall mean on technical performance score. The embedded participant S61 is considered as a helper because his/her influence on technical performance is higher than the overall mean of all the others EP. ns: non-significant: the influence of the facilitator is similar to all others (neutral embedded participant). ***: p value < 0.001

A mild influence of the EP on the technical score obtained by learners was observed (S = 35.8, df = 18, p = 0.008, eta^2^ = 0.055, Fig. 1). Three EPs demonstrated a trending influence on technical score (p < 0.010); their influence was associated with a decreased performance (distractors: D8, E9, P25). Compared to all other EPs, only one significantly influenced the technical performance; his/her influence was associated with an increased performance (helper: S61) (Table 1).

**Table 1:** Influence of the embedded participant on learner technical and non-technical performance. Statistical results of the post-hoc comparisons that allow to detect if the influence of each EP was different to the mean influence of all other EPs

EP identity	Technical performance	Non-technical performance
	p value	p value
**A7**	0.382	0.038*
**B8**	0.263	0.025*
**C8**	0.568	0.610
**D8**	**0.073** *¤*	0.110
**E9**	**0.085** *¤*	0.574
**F9**	0.300	0.562
**G11**	0.152	0.317
**H11**	0.753	0.286
**I11**	0.568	0.019*
**J12**	0.506	**0.001** **
**K14**	0.546	0.290
**L14**	0.384	0.432
**M18**	0.945	0.510
**N21**	0.959	0.122
**022**	0.915	0.208
**P25**	**0.080** *¤*	0.748
**Q28**	0.757	0.005**
**R47**	0.227	**< 0.0001** ***
**S61**	**< 0.001** ****	0.131

**Technical performance.** When a strong correction for multi-testing (Holm correction) was applied, the influence of S61 on technical performance remains statistically different from all the others (adjusted P value: 0.003)

**Non-technical performance.** When the correction for multi-testing was applied, the influence of J12 (adjusted P = 0.018) and R47 (adjusted P > 0.001) on non-technical performance remains statistically different from all the others. The influence of Q28 became trendy (adjusted P value: 0.085), while the influence of A7, B8 and I11 became non-significant (adjusted P value of 0.53, 0.43 and 0.30 respectively)

¤ trend: p value < 0.10, * p value < 0.05, **p value < 0.01, ***p value < 0.001

**Fig. 2:**
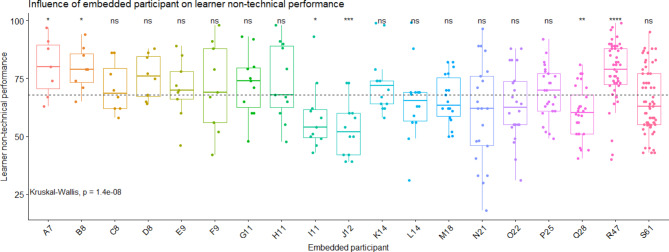
**Learner’s non-technical performance according to the embedded participant identity.** Each EP is presented with a code composed by a letter and a number, the number representing the number of participations in scenarios as embedded participant. Each point corresponds to one learner’s performance score. The grey dashed line corresponds to the overall mean on technical performance score. The embedded participants A7, B8 and R47 are helpers. I11, J12 and Q28 are distractors ns: non-significant: the influence of the facilitator is similar to all others (neutral embedded participant). * p value < 0.05, ** p value < 0.01, *** p value < 0.001

The influence of the EPs on the non-technical score obtained by learners was statistically significant (S = 73, df = 18, p < 0.0001, eta^2^ = 0.169, Fig. 2). Compared to all others, many EPs influenced the non-technical performance; three EPs increased the non-technical performance (helpers: A7, B8, R47) and three EPs decreased it (distractors: I11, J12, Q28) (Table 1).

### Influence of EP characteristics on performance

While the models did not show any effects of gender, previous training in simulation, expertise in simulation or teaching status of the EP, the results suggested an effect of the profession on technical performance and an influence of professional expertise on non-technical performance. For all regression models, β with its standard deviation (i.e., estimate the effect on the outcome of each 1-unit increase in the independent variable) and the intercept (the mean value of the response variable when all of the predictor variables in the model are equal to zero) are provided (Table 2). These trends suggest that learners’ technical performance was higher with non-physician EPs than with physician EPs (+ 4.17 ± 2.19, p = 0.077). They also suggest that shorter EPs’ professional experience was associated with higher non-technical performance of the learners (-0.46 ± 0.24, p = 0.095). The overall characteristics of the EPs according to their influence on learners is given in Table 3.

**Table 2:** Influence of the embedded participants’ characteristics on technical and non-technical performance (statistics of the linear mixed models)

		A. Technical performance				B. Non-technical performance			
EP characteristics		Intercept	Estimate	Std error	P value	Intercept	Estimate	Std error	P value
Gender	Men *versus* women	43.93 ± 3.56	-0.20	2.01	0.921	79.16 ± 5.76	-2.76	3.34	0.427
Training in simulation	Yes *versus* no		1.45	2.62	0.590		4.53	4.43	0.326
Expertise in simulation	Novice *versus* expert		-2.90	1.84	0.130		0.81	3.03	0.795
Teaching status	Yes* versus* No		-2.23	1.45	0.165		0.03	2.66	0.99
Profession	Non-physicians *versus* physicians		4.17	2.19	0.077		4.76	3.66	0.217
Expertise in profession	Continuous variable		-0.14	0.13	0.330		-0.46	0.24	0.095

**Table 3:** Characteristics of the embedded participants

		Helper	Distractor	Neutral
Age (years old)	30	1	1	2
	40	3	0	4
	50	0	1	2
	60	0	1	3
Gender	Women	1	1	4
	Men	3	2	8
Training in simulation	No	0	1	2
	Yes	4	2	10
Formation site	Lyon Healthcare Simulation	4	1	7
	Other	0	1	3
Type of training	University diploma	3	2	8
	Other	1	0	2
Experience in simulation	Expert	2	2	8
	Novice	2	1	4
Teaching status	Yes	1	1	3
	No	3	2	9
Profession	Physician	2	3	9
	Other	2	0	3
Experience in profession (years)	5	0	1	3
	10	4	0	5
	20	0	2	4

### Survey: inventory of the practices of EPs in France

A total of 147 responses to questionnaires were collected from 35 different simulation centres in mainland France, overseas territories of France, Switzerland, Belgium and Morocco between April and October 2021. The average age of the respondents was 46 (± 9, from 25 to 67) years old, with a slight majority of males (58%). The respondents were 52% doctors, 39% nurses, 5% other health professionals, and 4% non-medical professionals. Their average duration of exercise was 17 years (± 9, from 2 to 45). 90% of respondents had received training as an instructor in simulation, 76% obtained a university degree, and 66% declared a teaching activity outside the simulation. They were trained in 23 training centres throughout France (Appendix 2). Respondents rated the role of the EP as the second most difficult activity in an HFS session, behind “debriefing” and ahead of “command and control of the manikin” and “briefing”. Only 57% of respondents reported that an EP was always present in their HFS scenarios. A communication modality such as headset between the instructor and the EP during the scenario was used in 50% of cases. Rules of good practice defined by the French High Authority of Health concerning the attitude of the EP existed in approximately 36% of the centres. 82% of the EPs declared that they actively participated in the debriefing. They felt that the way they facilitated could also affect the quality of the debriefing and that it often led to focused discussions. The EPs wished they had more guidance from the lead instructor during the scenario. However, instructions on the behaviour to adopt were often given before the scenario. Without specific instructions, EPs reported most often adopting a helping role. Importantly, 65% of respondents felt that specific EP training would be warranted, while only 9% said they had received one.

## Discussion

This study aims to assess the influence of the EP on the technical and non-technical performance of learners during simulated situations. The present findings showed that EPs might influence the technical and non-technical performances of learners during HFS. It seemed that the EPs could exert either a positive or a negative influence on these performances. This effect appeared heterogeneous and not predictable by either the characteristics of the EPs or by their experience in simulation. However, there was a tendency for poorer performance when the facilitator was an experienced physician playing the role of a nurse. The survey highlighted a significant variability of practices and the lack of training concerning the role of the EP.

Standards of best practice for simulation have been widely reviewed and it is known that the performance and learning of participants depend on the immersion quality of a simulation scenario [8, 10]. Physical, or environmental fidelity specificallly relates to how realistically the context of the simulation-based activity simulates the actual environment in which the situation would occur in real life [8, 10]. Therefore, the use of an EP plays an important role tin maintaining this environmental fidelity and ensuring participants’ full immersion. Surprisingly, little attention has been given to the role of the EP in the literature. The current work is therefore the first to focus on the EPs and their influence on both technical and non-technical performance during HFS. EP’s profession, experience and other characteristics may play a role in the scenario that may affect the learners. Simulation instructor courses are becoming increasingly available through a variety of educational options. Setting up a simulation program, defining educational objectives for learners, the steps of building a scenario, and the key stages of a briefing and debriefing sessions are subjects that are deeply expanded through trainees [14]. With the number of fundamental steps that need to be learned, the EP and his role are most of the time vaguely explored. Previously, in a study conducted in 2015, when the top elements that potential instructors wish to learn in simulation training courses were asked, the role of the EP was considered the least important [15]. Controversially, in 2022, the respondents of our survey estimated the role of the EP as the second most difficult and mentioned that a specific EP training could be beneficial.

The EP often enriches immersive healthcare simulation scenarios. According to our results, only one EP (S61), who was not a physician, had a positive influence on learners’ technical performance. Considering that the role of the EPs in all scenarios was to play the “assisting nurse”, a physician playing this role is more likely to show medical skills during the scenario than a nurse playing his/her own role. Lacking any EP-specific training, there might be some disruptive attitude of the physician playing the EP that is catched by the learner and results in disturbance of learner immediate performance. Is it inoportune initiatives, feelings of higher expectations from the EP, less fluidity in nurses’ behaviours, or the fact that the EP is already known as a real physician that are factors providing these effects on performance? There is a need for further clarification studies to explore those hypotheses. These observations reinforce the idea that an EP should ideally play his/her own profession role for more realism.

Six EP influenced the learner’s non-technical performance. Some were distractors and others were helpers. EPs with long professional experience seemed to be associated with poorer non-technical performance of learners. One might suspect that experience could come with age, and the greater the age of the EP, the greater the learner feels unconfortable with keeping the leadership, which results in a decrease in non-technical objective performance. Once again, further studies will certainly bring rational explanation to that observation as it may result in pedagogical impact and/or summative impact if simulation is becoming an assessment tool. Previous literature has shown that clinical experience is not associated with effectiveness as a simulation instructor [15, 16]. Lee et al. demonstrated that junior staff doctors felt more comfortable in HFS facilitation and similarly, years of clinical experience did not increase their comfort in teaching HFS [15]. Moreover, a relationship between teachers’ personality and teaching effectiveness has been demonstrated [17]. Personality provides a behavior that reflects human interactions, and it seems more difficult to modify an individual’s technical knowledge than to help him/her express the non-technical skills. Therefore, it seems easily understable that the EPs influenced the non-technical performances more than the technical performance. Therefore, we may suggest that the performance of learners is affected not only by demographic factors such as age, gender, or experience, but also by other factors such as emotional intelligence, personality of the EP, gender mix and human interactions between both the learner and the EP [18]. Among the few studies focusing on the EP, one explored the effect of the presence of an EP on participants’ non-technical performance during simulated practice of medical crisis situations [19]. In contrast to our results, a previous study conducted by Traoré et al. reported that the presence of EPs during simulation scenarios did not seem to improve the crisis resource management skills of learners [19]. They presented a non-significant difference when comparing overall crisis resource management performance between learners who were accompanied by an EP and those who were not. These results are controversial with our findings that suggest a greater influence of the EP on non-technical performance than on technical performance. Previously, Mavis et al. evaluated the impact of standardized patients on students’ experiences by comparing faculty members and students to portray this role [20]. They demonstrated that faculty member standardized patients provided more helpful feedback to improve skills even if the students mentioned they were more intimated. Students were less anxious when the EP was a peer but they described the simulation session as less valuable [20]. According to the clinical environment, faculty members with related expertise may lead to a rich clinical experience. However, our results did not show any significant effect of the teaching status of the EPs, yet this could be due to their small number and to the diversity of EPs’ characteristics.

### Strengths and limitations

To our knowledge, this study is the first one to highlight a potential effect of the EPs on learners’ both technical and non-technical performance. While an important number of simulation scenario were reviewed (more than 300), several limitations can be underlined. First, the effect of 19 EPs was analysed: although this number may be seen as important in terms of training and pedagogy, it is still modest regarding statistics. A larger sample size of EPs might have allowed to highlight the main characteristics that could lead to impact on learners’ performance. Second, the EPs did not facilitate an equal number of scenarios and the scenarios did not exhibit the same level of difficulty. However, the retrospective nature of the study over a period of five years could have brought heterogeneity to the EP population since EPs with different professions and backgrounds were involved. Third, residents may have gone through several consecutive simulation sessions, and may have encountered the same EP when performing as active participant. However, the active participants were anonymized with no correspondence table between years and not possible to identify with certitude from the voice, the face covered by surgical mask and hat. The authors believe that this eventuality would not influence the conclusions of the study. Fourth, the performance of the learners was evaluated according to the taken actions. When an action considered essential to the scenario was not performed, the EP suggested it implicitly or explicitly, on instructor request through the headset. This resulted in a decrease in the performance score. Further studies analysing directly the behaviour should be considered. This is indeed one of the major points of this study and underlines how an EP can influence the performance of the participant. Fifth, the performance of the learners was evaluated according to the taken actions. When an action essential to the scenario was not performed, the EP suggested it implicitly or explicitly, resulting of a decrease in performance score. Further studies analysing directly the behaviour of the EP should be considered. This highlights the importance of this study and how an EP of a simulation scenario can influence the performance of the learners. Sixth, no evaluation was performed after the simulation. Simulation is first a pedagogical tool. Therefore, it might be more interesting to explore the effect of some facilitation compared to others in the subsequent simulation performance of in further real performance of learners. Finally, the simulation as a formative tool is not comparable to simulation as a summative tool for the assessment of students or professionals. The objective structured clinical examination (OSCE) as a specific simulation assessment tool shall be extensively explored for all interactions between standardized patient and student that may affect performance of the student. The impact of the success or failure on the OSCE is major for student curricula. Therefore, the impacts of uncontrolled interactions between EPs and students should be further explored and the positive or negative influence of the EP on the student’s performance should be addressed to avoid the risk of the tool being discredited by students [21]. The present study could be considered an incentive for more clarification studies to address these questions.

## Conclusion

In HFS, the EP and his/her behavior seem to influence learners’ performance. While the effect on technical performance remains small, our findings underline that EPs’ influence on non-technical performance is significant. Characteristics of the EPs such as profession and expertise may also have an influence on learners’ performance. This effect seems heterogeneous and appears to stem from behaviour rather than from demographic characteristics or experience. Future studies highlighting the influence of the EP should include emotinal intelligence, personality and specific interaction analysis. The survey sent to simulation centres highlights a great variability of practices and the lack of training dedicated to the role of the EP. These results suggest paying more attention to the preparation and training of EPs and underline the need for future research focused on the interaction between the EP and the HFS session especially as simulation is extensively used as an assessment tool.

### Electronic supplementary material

Below is the link to the electronic supplementary material.

Supplementary Material 1

Supplementary Material 2

## Data Availability

The datasets and materials used and/or analyzed during the current study available from the corresponding author on reasonable request.
