# Supplementary material for: Influence of the embedded participant on learners’ performance during high-fidelity simulation sessions in healthcare
Source: BMC Med Educ. 2023 Oct 11;23:751. doi: 10.1186/s12909-023-04724-0 (PMC10568852; doi:10.1186/s12909-023-04724-0)
Supplement: Supplementary file 1 — Supplementary Material 1 [file 12909_2023_4724_MOESM1_ESM.docx]

**Additional file 2. Survey - The role of the embedded participant in high-fidelity simulation**

This questionnaire aims to investigate the role of the embedded participant during computer-controlled manikin high-fidelity healthcare simulation.

By high-fidelity healthcare simulation we mean formative (and not summative) simulation session scheduled in advance with notice, session including simulation briefing, scenario briefing, scenario without interruption then individual or collective debriefing. Simulation sessions with feedback or debriefing during the scenario (within-event debriefing) are not considered.

By the term “embedded participant” (EP), we mean the definition from the Healthcare Simulation Dictionary (https://www.ssih.org/dictionary): “Definition • An individual who is trained or scripted to play a role in a simulation encounter in order to guide the scenario, and may be known or unknown to the participants; guidance may be positive or negative, or a distractor based on the objectives, level of the participants, and the needs of the scenario. • A role assigned in a simulation encounter to help guide the scenario. The embedded participant's role is part of the situation. However, the underlying purpose of the role may not be revealed to the participants in the scenario or simulation (INACSL, 2013).”

If you have already participated in this type of high-fidelity healthcare simulation by taking on this role, please complete this survey.

If you do not wish to participate in this research after reading the information above, please close the webpage.

If you are willing to participate in the research, continue by selecting the "Yes" option.

YES

**SECTION 1: GENERAL INFORMATION**

1. What is your name, surname?
2. How old are you?

- 18-25
- 25-35
- 45-55
- 55-65
- >65

3. What is your gender?

- Man
- Woman
- Other

1. What is/are your profession(s)?
2. How many years have you been working in this profession?
3. Do you have formal training as a simulation instructor?

Yes - No

1. If yes, in which simulation center have you been trained?

8. If yes, do you have a teaching activity apart from the simulation center?

Yes - No

**SECTION 2: YOUR HİIGH-FİDELITY SIMULATION INSTRUCTOR EXPERIENCE**

1. In which simulation center do you practice?
2. How long (years or months) have you been participating in high-fidelity simulation sessions?

- Less than 6 months
- 6 months-1 year
- 1-2 years
- 3-5 years
- More than 5 years

1. In your opinion, as an instructor, how difficult are the different parts of a high-fidelity simulation session?


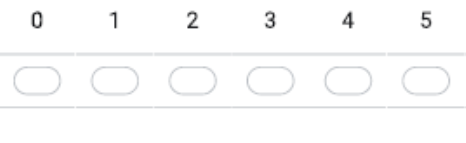


(0: very easy / 5: very difficult)

- Briefing
- Manikin control
- The role of the EP
- Debriefing

4. Within your simulation centre, do you have a system/technology (e.g., headset) allowing the instructor in charge to communicate information to you live and in private?

Yes – No

5. Is there always an EP during your scenarios?

Yes - No

6. Do you attend the debriefing after being the EP of a scenario?

Yes - No

1. Do you actively participate in debriefing after being the EP of a scenario?

Yes - No

1. Is there a rule of good practice clearly established by the simulation center concerning the attitude of the EP during the scenario?

Yes – No

1. If yes, please specify:

**SECTION 3**

Please answer the following questions considering your role as an EP during a HFS session


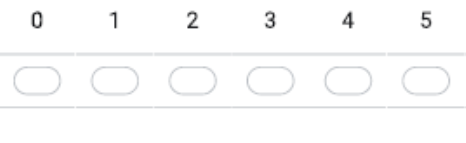


1. Are you usually guided by the responsible instructor during the scenario?

0: no never - 5: yes always

1. Would you like to be guided by the instructor responsible for the scenario during your role as a EP?

0: no never - 5: yes always

1. Is an instruction on the behavior of the EP clearly explained to you before the start of the scenario?

Yes - No

1. For educational purposes, and without specific instructions concerning your behavior, what behavior do you adopt?

0: Rather Negative, Disruptive – 5: Rather Positive, Helping

1. Do you think specific EP training is warranted?

Yes - No

1. Did you attend a specific EP training?

Yes - No

**APPENDIX 2. Holm correction Influence of the embedded participant on learner technical and non-technical performance. Statistical results for the *post-hoc* comparisons**

| **EP identity** | **Technical performance** | **Non-technical performance** |
| --- | --- | --- |
|  | **Adjusted p value** | **Adjusted p value** |
| **A7** | 1 | 0.530 |
| **B8** | 1 | 0.437 |
| **C8** | 1 | 1 |
| **D8** | 1 | 1 |
| **E9** | 1 | 1 |
| **F9** | 1 | 1 |
| **G11** | 1 | 1 |
| **H11** | 1 | 1 |
| **I11** | 1 | 0.3 |
| **J12** | 1 | **0.018*** |
| **K14** | 1 | 1 |
| **L14** | 1 | 1 |
| **M18** | 1 | 1 |
| **N21** | 1 | 1 |
| **022** | 1 | 1 |
| **P25** | 1 | 1 |
| **Q28** | 1 | 0.085 ^.*^ |
| **R47** | 1 | **<0.001***** |
| **S61** | **0.003**** | 1 |

**trend: p value < 0.10, ** p value <0.05, ** p value <0.01, *** p value <0.001.*

**APPENDIX 3. Demographics of the survey respondents**

| Sex | n(%) |
| --- | --- |
| Men | 85(57.8) |
| Women | 62(42.2) |
| Simulation centre |  |
| CLESS | 56(38) |
| Others | 91(61) |
| Age ranges |  |
| 25-35 years | 17(12) |
| 35-45 years | 60(40) |
| 45-55 years | 42(29) |
| 55-65 years | 27(18) |
| More than 55 years | 1(1) |
| Profession |  |
| Physician | 77(53) |
| Midwife | 4(2.6) |
| Pharmacist | 1(0.7) |
| Nurse | 57(39) |
| Physiotherapist | 1(0.7) |
| Other | 6(4) |
| Simulation instructor training |  |
| Yes | 132(90) |
| Master | 3(2) |
| University degree | 103(78) |
| Masterclass | 15(11) |
| Short training | 11(8) |
| No | 15(10) |
| HFS experience |  |
| Less than 1 year | 7(5) |
| 1-2 years | 18(12) |
| 3-5 years | 24(16) |
| More than 5 years | 98(67) |

*CLESS: Lyon Healthcare Simulation Centre, HFS: High-fidelity simulation*
