# Supplementary figures and images for: Influence of the embedded participant on learners’ performance during high-fidelity simulation sessions in healthcare
Source: BMC Med Educ. 2023 Oct 11;23:751. doi: 10.1186/s12909-023-04724-0 (PMC10568852; doi:10.1186/s12909-023-04724-0)

Additional file1. Evaluation grid for the epiglottis scenario


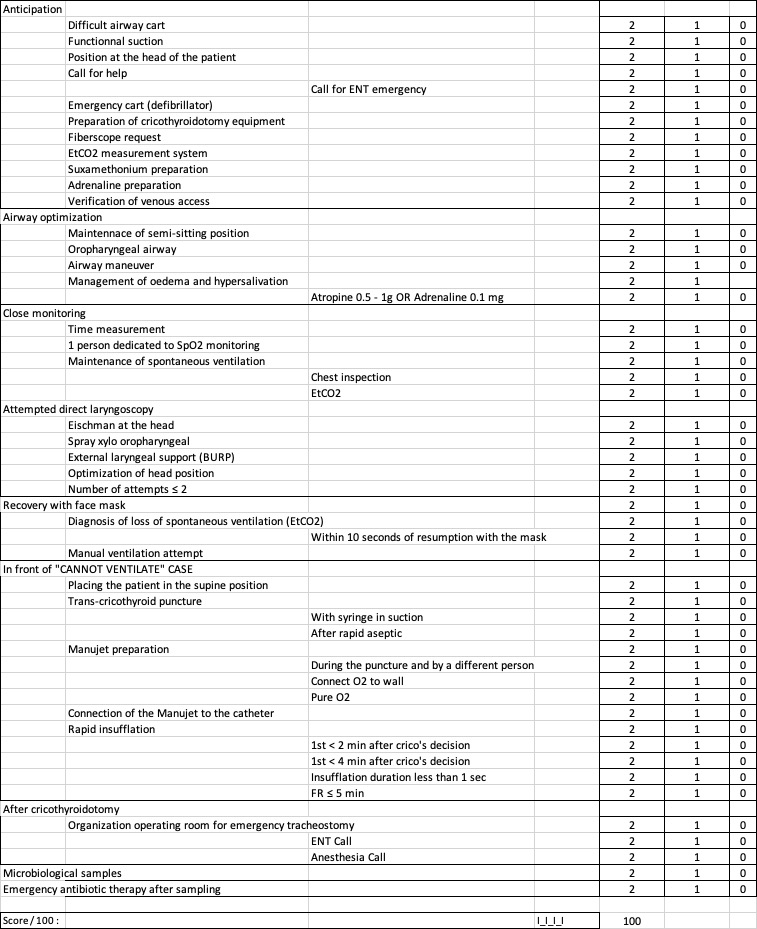

Supplement: Supplementary file 2 — Supplementary Material 2 [file 12909_2023_4724_MOESM2_ESM.docx]
